# Candida Costochondritis Induced by Traumatic Small Bowel Perforation: A Case Report

**DOI:** 10.7759/cureus.43923

**Published:** 2023-08-22

**Authors:** Hideharu Nakamura, Takaya Makiguchi, Aya Tsunoda, Ken Shirabe, Satoshi Yokoo

**Affiliations:** 1 Department of Plastic and Reconstructive Surgery, National Hospital Organization Takasaki General Medical Center, Takasaki, JPN; 2 Department of Oral and Maxillofacial Surgery, Plastic Surgery, Gunma University Graduate School of Medicine, Maebashi, JPN; 3 Department of General Surgical Science, Gunma University Graduate School of Medicine, Maebashi, JPN

**Keywords:** abdominal surgery, trauma, candida costochondritis, candida osteomyelitis, invasive candidiasis

## Abstract

Candida osteomyelitis is a manifestation of invasive candidiasis. The common sites of infection are the vertebra, sternum, and femur, whereas infection of the rib cartilage is very rare. In the present case, candida costochondritis developed after traumatic small-bowel perforation. An 82-year-old man was involved in a traffic accident while walking. His past medical history was insignificant. He was diagnosed with a pelvic fracture and perforation of the small intestine and underwent open reduction and internal fixation of the pelvic fracture and an enterectomy. Three months after the injury, swelling was observed in the right anterior thoracic region. Swelling was treated by incision and drainage but persisted in the form of infected granulation tissue. Debridement, including rib cartilage removal, was done. Biopsy and culture of the removed granulation tissue and cartilage tissue confirmed candida costochondritis. Fluconazole was administered for six months. No recurrence has been observed in the seven months postoperatively. Candida costochondritis is rare but is often refractory and requires extensive debridement in addition to the administration of antifungal agents. This disease should be included in the differential diagnosis when pain, erythema, swelling, skin ulceration, or infected granulation is found on affected costal cartilages.

## Introduction

Candida species have a high affinity for humans and are ubiquitous on mucous membranes and skin. Superficial candidiasis, such as oropharyngeal candidiasis, vulvovaginal candidiasis, and candida dermatitis, is common in daily practice. Invasive candidiasis comprises both candidemia and deep-seated-tissue candidiasis and has the major risk factors of central vascular catheters, surgery, and broad-spectrum antibiotic therapy [1]. Candida osteomyelitis is a manifestation of invasive candidiasis. The common sites of infection are the vertebra, sternum, and femur, whereas infection of the rib cartilage is very rare [2]. Candida costochondritis has been described in association with an injectable drug (heroin), but there have been few reports in recent years. [3]. In the present case, candida costochondritis occurred after a small bowel perforation associated with traffic trauma.

## Case presentation

An 82-year-old man was involved in a traffic accident while walking and was taken to the hospital. His past medical history was insignificant. He was diagnosed with a pelvic fracture and perforation of the small intestine and underwent open reduction and internal fixation of the pelvic fracture and an enterectomy. After surgery, the patient received systemic treatment in the intensive care unit and was transferred to the general ward on the 12th postoperative day. Tazobactam/piperacillin (13.5 g/day) was administered for nine days from the day of surgery, followed by meropenem (3.0 g/day) for 10 days. At this time, computed tomography (CT) showed no damage or mass formation in the rib cartilage.

Three months after the injury, the patient's general condition improved, and he was transferred to another hospital for rehabilitation. After transfer, a skin mass developed on the right anterior thoracic region. An inflamed epidermal cyst was suspected, and incisional drainage was performed, but the mass did not improve.

**Figure 1:**
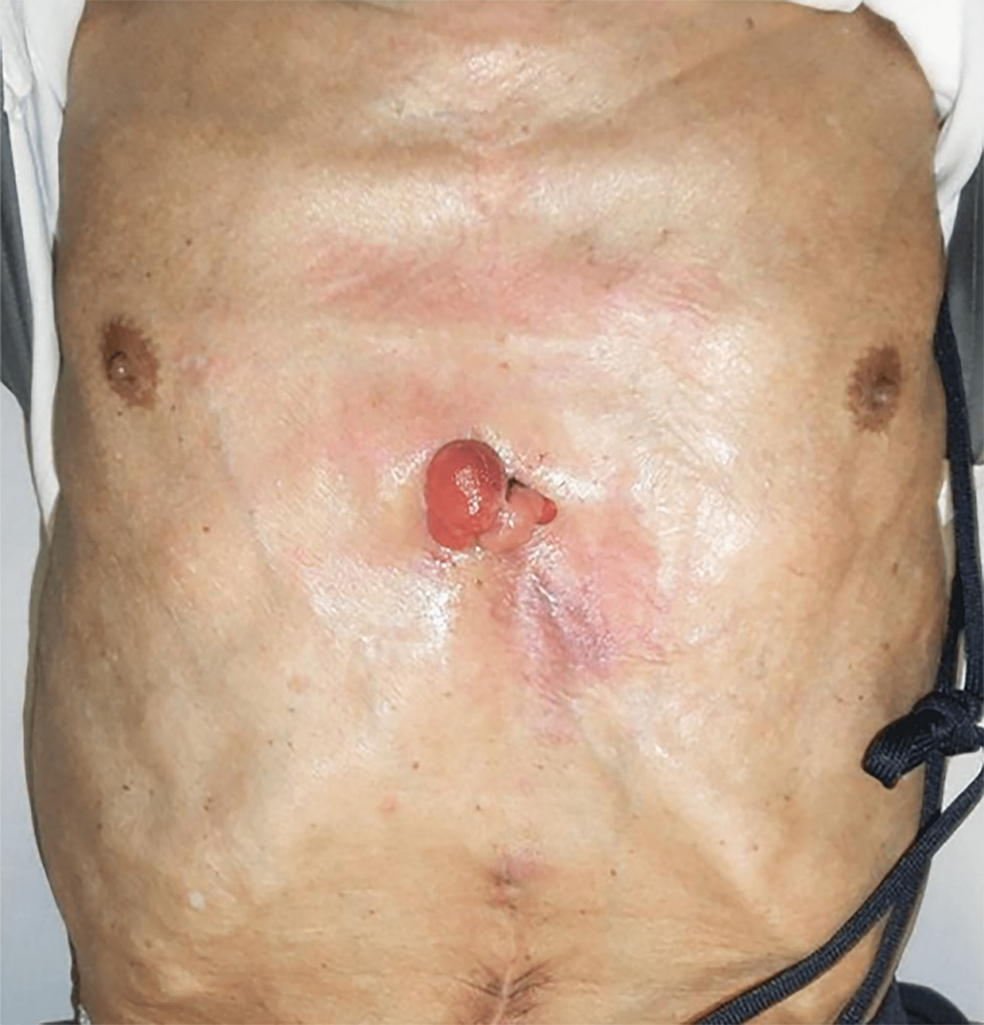
Chest findings at the initial examination. A 5-cm infected granulation tissue growth on the right anterior thoracic region was draining pus.

**Figure 2:**
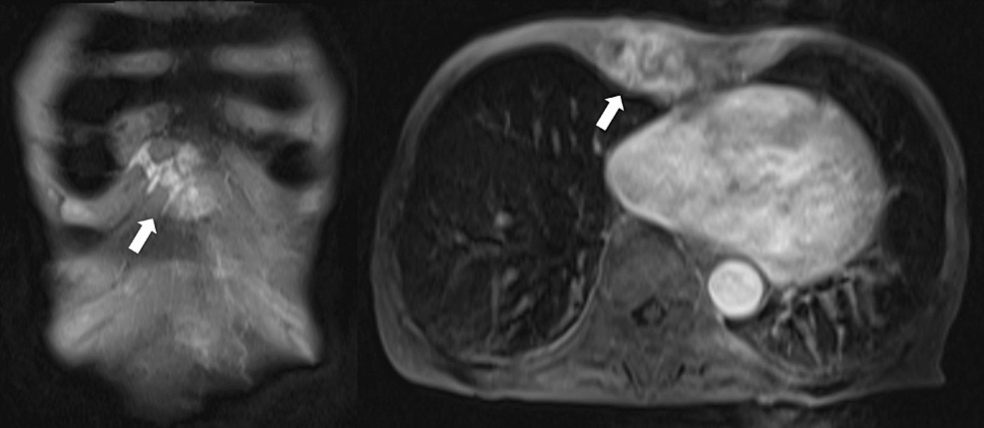
Chest MRI findings. MRI showed melting images of the right fifth, sixth, and seventh costal cartilages and surrounding mass formation (white arrow).

Five months after the injury, the patient was back at our hospital. At the time of transfer, there was a 5-cm infected granulation tissue growth on the right anterior thoracic region, which was draining pus (Figure 1). Tenderness was noted on the costal cartilage around the infected granulation, and there was erythema of the surrounding skin. The wound culture of the area identified Candida albicans. Laboratory tests showed a high level of β-D-glucan of 20.4 pg/ml (reference value: <11 pg/ml). White blood cells (WBCs) were in the normal range of 5,600 /µL (reference range: 4,000-9,600 /µL), and C-reactive protein (CRP) was mildly elevated at 2.19 mg/dL (reference range: <0.1 mg/dL). MRI showed melting images of the right fifth, sixth, and seventh costal cartilages and surrounding mass formation (Figure 2). There were no lesions other than the rib cartilage.

First surgery

**Figure 3:**
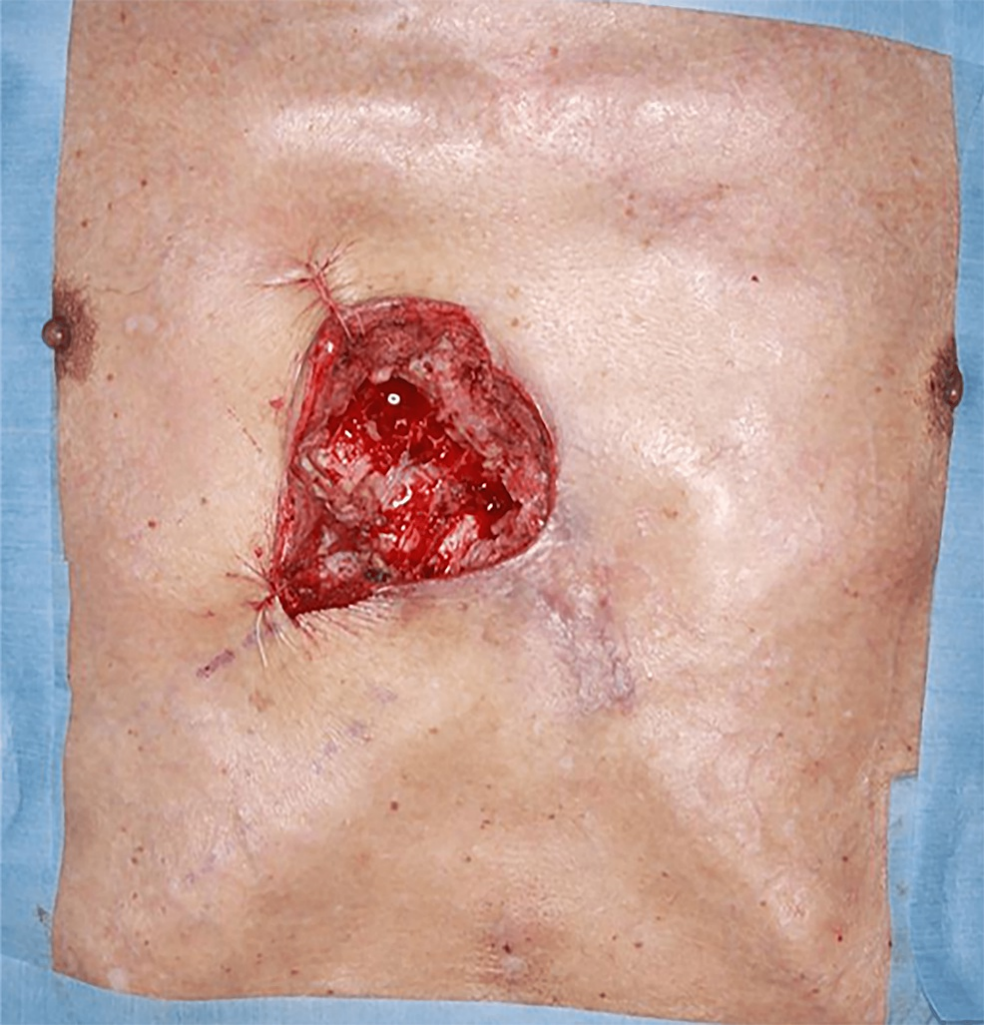
First surgery (debridement). The infected granulation tissue was resected, and the fifth, sixth, and seventh costal cartilages were partially removed.

Debridement was performed under general anesthesia. The infected granulation tissue was resected, and the fifth, sixth, and seventh costal cartilages were partially removed because cartilage lysis and sequestrum were observed (Figure 3). Given the risk of recurrence, the wound was not closed but left open temporarily, and wound closure was planned when granulation tissue growth was observed.

Histopathological examination

**Figure 4:**
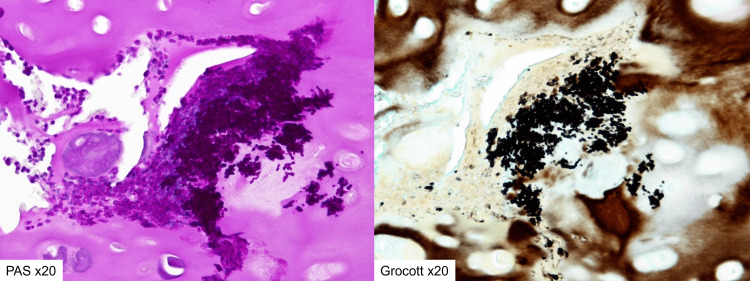
Histopathological examination Histopathological examination revealed a cluster of neutrophils in the cartilage. PAS staining and Grocott staining were positive.

Histopathological examination of the rib cartilage tissue removed during surgery revealed a cluster of neutrophils in the cartilage and positive PAS staining and Grocott staining in the same area (Figure 4). In addition, Candida albicans was detected in a culture test of the removed granulation tissue and cartilage tissue. Candida costochondritis was diagnosed, and fluconazole 250 mg/day (6 mg/kg) was started.

Second surgery

**Figure 5:**
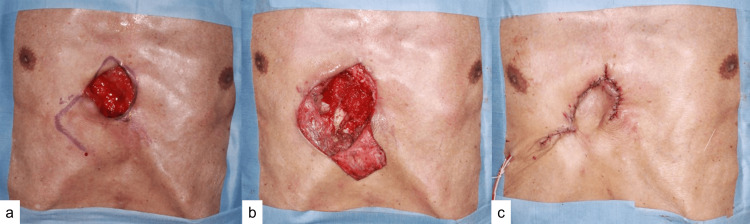
Second surgery (wound closure). (a) Preoperative finding. Local flap design. (b) After debridement and flap elevation. (c) After closing the surgical incision.

**Figure 6:**
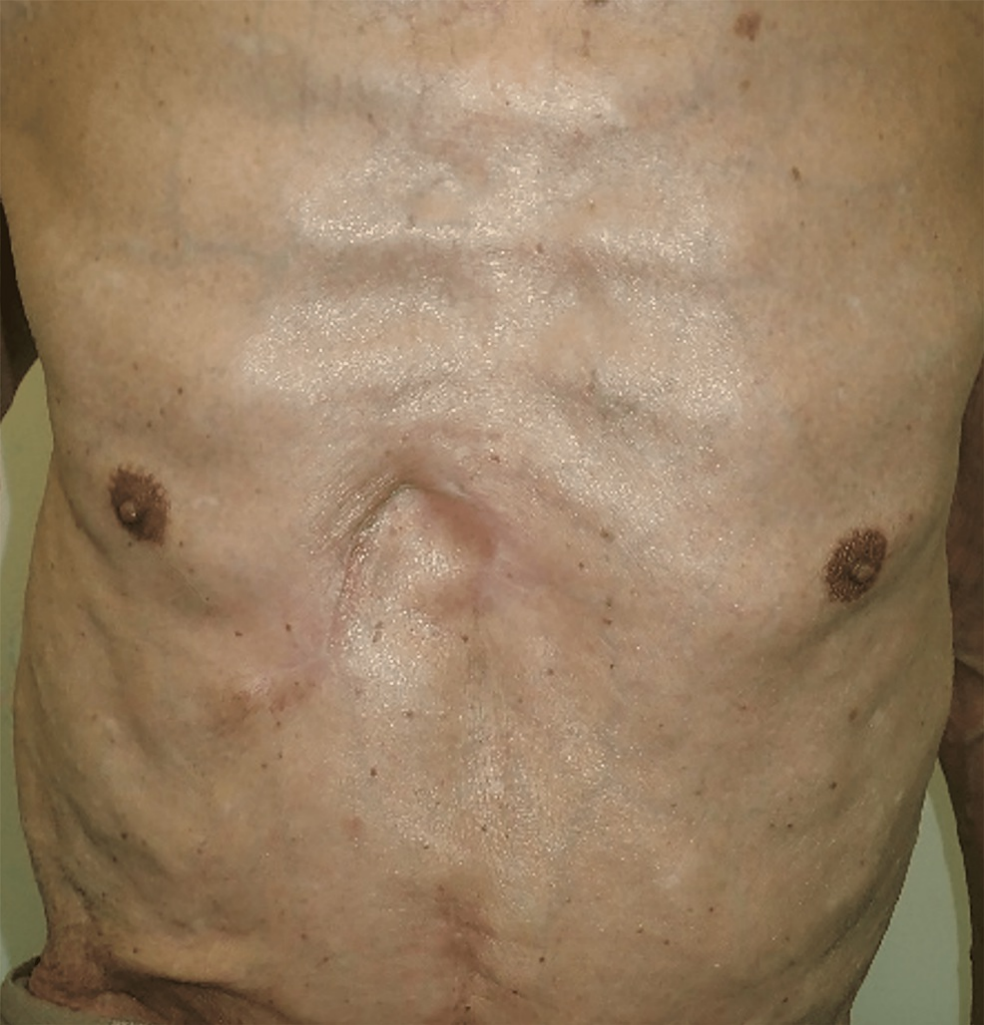
Chest findings at seven months postoperatively. No recurrence was observed.

After continuation of conservative treatment and observation of good granulation tissue growth, wound closure with a local flap (Limberg flap) was performed under general anesthesia four weeks after the first surgery (Figure 5). The postoperative course was uneventful, and the patient was transferred for rehabilitation 15 days after the second surgery. Fluconazole was continued for six months, and no recurrence has been observed at seven months postoperatively (Figure 6).

## Discussion

Invasive candidiasis comprises both candidemia and deep-seated-tissue candidiasis, which arises from hematogenous dissemination or direct inoculation of Candida species at a sterile site. Central venous catheterization, abdominal surgery with anastomotic leakage, and broad-spectrum antimicrobial therapy are the main risk factors for invasive candidiasis. Once candidiasis develops, fungi may disseminate and cause secondary metastatic infections in the lungs, liver, spleen, kidneys, bones, and eyes [1].

Candida osteomyelitis is a manifestation of invasive candidiasis, with common sites of infection in the vertebra (64%), sternum (25%), femur (4%), hips (7%), facial bone (3%), foot/ankle (3%), and tibia (2%) [2,4]. Candida costochondritis has been reported in association with an injectable drug (heroin), but there have been few reports in recent years due to changes in drug availability and injection practices [5]. Other cases have been related to surgeries such as thoracic surgery [6], esophagectomy [7], and trauma [8]. In our case, the patient had intestinal perforation due to traffic trauma and underwent small intestine resection, which may have led to candidiasis caused by Candida albicans, resulting in secondary metastatic infection of the rib cartilage. Localized symptoms include pain, erythema, and swelling on the affected costal cartilages [9], and skin ulceration and infected granulation may occur [7]. A high fever is rare, and WBCs and CRP are often only mildly elevated. Candida spp. is rarely detected in blood culture, and fungal infections are confirmed by culture of debrided tissue [3].

Heckenkamp et al. suggested that X-rays do not reveal costochondritis in the early stages, that ultrasonography may contribute to the detection of soft tissue lesions such as diffuse swelling of costal cartilages and abscess formation, and that MRI is superior to CT for detecting cartilaginous damage [8]. Gamaletsou et al. found that 44% of patients with candida osteomyelitis were treated with antifungal agents alone, 5% with surgery alone, and 48% with a combination of antifungal and surgical therapy [10]. The spine is rich in blood flow and may benefit from antifungal therapy, whereas the costochondral region is often difficult to treat with conservative treatment alone due to poor blood flow limiting antifungal transfer, and extensive surgical resection is often necessary.

For wound closure after debridement for osteomyelitis, well-vascularized tissue coverage is reported to prevent secondary infection by contamination and promote wound healing [9,11]. In addition to the rhomboidal flap in the present case, a rotation flap has been reported as a local flap for Costochondritis. The first choice for a pedicle flap would be a pectoralis major, latissimus dorsi, or rectus abdominis muscle flap [9]. The use of an appropriate flap for wound closure is recommended, depending on the extent of the defect and the condition of the wound.

Oral fluconazole (6 mg/kg) daily for 6 to 12 months or echinocandin 50 to 70 mg intravenously daily for at least two weeks, followed by oral fluconazole (6 mg/kg) daily for 6 to 12 months, is recommended as antifungal therapy for Candida osteomyelitis by the Infectious Diseases Society of America [12]. In our case, fluconazole (6 mg/kg) was administered for six months in addition to surgical debridement, and the patient had no recurrence and is doing well.

## Conclusions

We report a case of candida costochondritis following traffic trauma. Candida costochondritis has been described in association with an injectable drug (heroin) and surgery but can also occur after severe trauma, as in this case. Candida costochondritis is rare but is often refractory and requires extensive debridement in addition to the administration of antifungal agents. This disease should be included in the differential diagnosis when pain, erythema, swelling, skin ulceration, or infected granulation is observed on affected costal cartilages.
